# Is Problematic Internet and Smartphone Use Related to Poorer Quality of Life? A Systematic Review of Available Evidence and Assessment Strategies

**DOI:** 10.1007/s40429-022-00415-w

**Published:** 2022-06-11

**Authors:** Nassim Masaeli, Joël Billieux

**Affiliations:** 1 South Tehran Branch, Islamic Azad University, Tehran, Iran; 2 Institute of Psychology, University of Lausanne (UNIL), Lausanne, Switzerland; 3 Centre for Excessive Gambling, Addiction Medicine, Lausanne University Hospitals (CHUV), Lausanne, Switzerland

**Keywords:** Problematic Internet use, Problematic smartphone use, Quality of life, Health-related quality of life, Systematic review

## Abstract

**Purpose of Review:**

Previous studies have explored the links between problematic Internet use (PIU) or problematic smartphone use (PSU) and quality of life (QOL). In this systematic review, we (i) describe the instruments used to assess QOL or health-related quality of life (HRQOL) in these studies, (ii) critically examine the content validity of the instruments used, and (iii) examine the relationships between PIU, PSU, QOL, and HRQOL.

**Recent Findings:**

We identified 17 PIU and 11 PSU studies in a systematic search. Evidence suggests that PIU and PSU negatively correlate with either QOL or HQOL and most of their domains (especially mental and physical health). Multiple instruments were used to assess QOL or HRQOL in these studies. Our analysis showed an important heterogeneity in the domains covered by these instruments.

**Summary:**

Because of the widespread prevalence of PIU and PSU, which tend to be linked with lower QOL or HRQOL, in particular poor mental and physical health, a more systematic public health campaign is required to target the healthy use of these communication devices. Prevention programs should also target vulnerable individuals, focusing on the most affected domains of QOL and HRQOL (i.e., physical and psychological health). Among the existing instruments, the World Health Organization Quality of Life for adults and the Pediatric Quality of Life Inventory for adolescents (aged 13–18 years) proved to be the most relevant, although new measurement instruments are needed to target domains that are specifically relevant in the context of PIU and PSU (e.g., physical and psychological health domains such as sleep, loneliness, and quality of familial relations).

## Introduction

The use of the Internet and smartphones has become a global phenomenon. Digital technology advancements have resulted in a wide range of applications, including improved communication, health, education, and leisure. Nonetheless, during the last two decades, a growing number of studies reported links between problematic or uncontrolled digital technology use and various indices of psychological and health problems [1–3]. Moreover, for a minority of vulnerable persons, excessive use of online applications (such as online video games, online sexual activities, on-demand streaming platforms, and social network sites) can become problematic and engender negative consequences and functional impairment [4, 5].

Internet and smartphone-mediated problematic online behaviors have been conceptualized within a spectrum of related conditions associated with both shared and unique features and risk factors [6, 7]. It was also proposed that Internet use disorders should be considered according to the devices used (i.e., mobile versus non-mobile devices), as some online activities are mainly performed through one type of device (e.g., instant messaging services like WhatsApp), whereas other online activities can be performed through both mobile and non-mobile devices (e.g., videogames) [8]. Accordingly, PIU and PSU overlap to some degree. Behavioral problems associated with the problematic use of digital technologies are often conceptualized as addictive disorders within a biomedical framework [9–10], although competing etiological models have been proposed. In particular, it has been suggested that these problematic behaviors can reflect impulse-control or obsessive-compulsive disorders, or constitute maladaptive coping displayed to regulate negative mood states or to face conditions such as anxiety or mood disorders [12••, 13].

Previous research has shown that problematic Internet use (PIU) and problematic smartphone use (PSU) are negatively associated with global life satisfaction [14, 15] and health-related quality of life (HRQOL) [16, 17]. However, this literature merely focused on determining prevalence rates and correlates (e.g., psychosocial variables) of “addictive” patterns of use (e.g., associated with symptoms of loss of control, or with tolerance-like or withdrawal-like symptoms). Indeed, previous studies often reported prevalence rates for various online problematic behaviors without considering whether the targeted condition was or was not associated with negative consequences or functional impairment. This issue is especially relevant in the context of technology use, which has become ubiquitous, and ignoring it risks pathologizing normal behavior or intensive but healthy usage patterns [12••, 18••], as reflected by the elevated prevalence rates often reported in the literature (e.g., exceeding 5% or even 10% in some cases). When more stringent criteria are applied and negative consequences or functional impairment are taken as a prerequisite to diagnose the condition, the reported prevalence rates diminish (e.g., 1–2% for problematic online gaming [19]).

As the majority of investigators who studied PIU and PSU in previous work did not take into account related negative consequences or functional impairment, we decided to systematically review the available evidence regarding the relationships between these problematic behaviors and quality of life (QOL), assuming that the presence of problematic or pathological behavior would be associated with poor QOL. The World Health Organization defines QOL as “an individual’s perception of their position in life concerning their goals, expectations, standards, and concerns in the context of the culture and value systems in which they live [20].” It is a broad concept influenced in a complex way by a person’s physical health, psychological state, level of independence, social relationships, and relationship to key features of their environment [21]. Instruments that assess QOL can be divided into (i) general QOL instruments that do not specifically focus on the subjective health state and (ii) instruments that assess HRQOL that classically focus on four specific domains: physical, physical well-being, psychological state, and social relations [22].

Several definitions of HRQOL have been proposed [23•], and in the present review, we consider HRQOL to reflect aspects of self-perceived well-being and perceived physical and mental health that are related to or affected by the presence of disease or treatment [24]. In contrast, QOL corresponds to the subjective feeling of satisfaction about important life domains [24]. The terms HRQOL and QOL are frequently used interchangeably [23•], and the medical literature has debated how to conceptualize and measure HRQOL since the 1960s [25], as it is a complex construct with no universally accepted definition [26]. However, it is agreed that it should not be defined as the absence of disease or disorder, but rather from a more holistic perspective that includes physical, psychological, emotional, and social factors.

In the present systematic review, we thus (i) describe the instruments used to assess QOL or HRQOL in PIU and PSU research, (ii) critically examine the content validity of the instruments used in these studies, and (iii) examine relationships between PIU, PSU, QOL, and HRQOL.

## Method

### Inclusion Criteria

We followed the Preferred Reporting Items for Systematic Reviews and Meta-Analyses (PRISMA) statement guidelines for systematic reviews [27, 28]. The inclusion criteria for eligible studies in the present systematic review were as follows: (i) studies published in scientific journals from 2011 to December 2021 (a 10-year period was considered to increase the potential number of studies included in this systematic review), (ii) studies written in English, and (iii) studies reporting the association between QOL or HRQOL and PIU or PSU. Moreover, studies that focused on specific online activities (e.g., social network use, online gambling, video gaming) were excluded, as the present review focused on the broader PIU and PSU constructs.

### Search Strategy and Study Selection

In the literature search, we aimed at identifying original empirical studies that reported correlations between PIU or PSU and QOL or HRQOL in the electronic databases Science Direct, PsycNET, and PubMed.

**Fig. 1:**
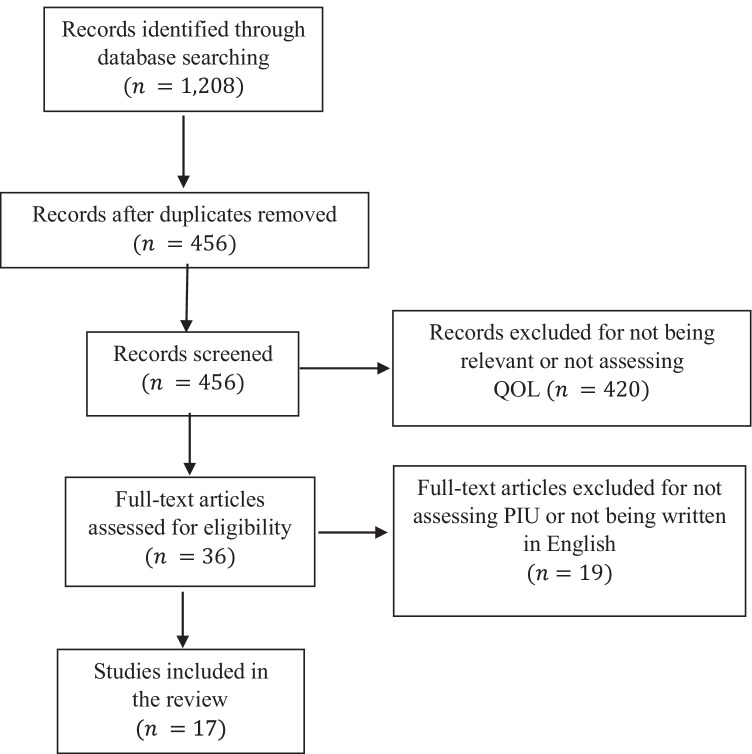
Flowchart for PIU studies

**Fig. 2:**
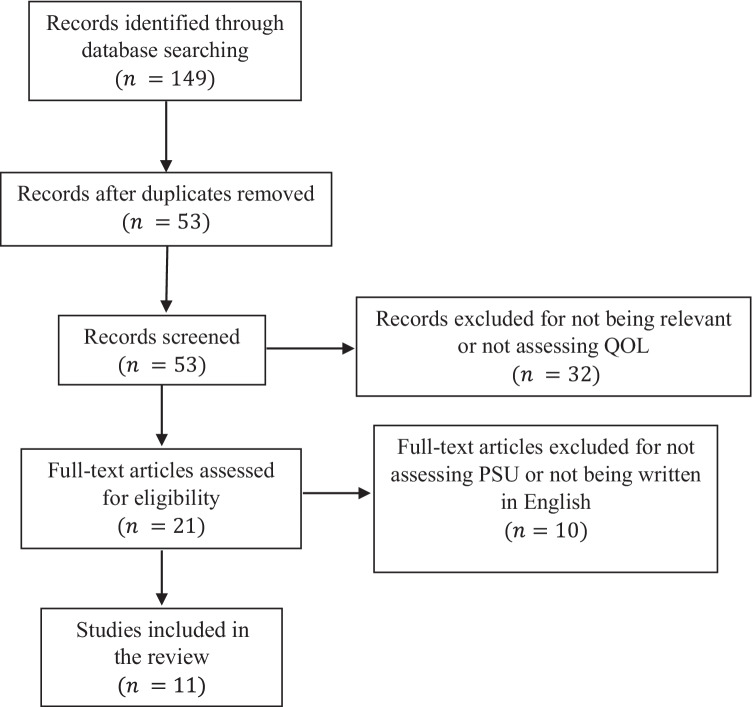
Flowchart for PSU studies

Two systematic literature searches were performed, one for PIU and one for PSU. Regarding PIU, the following terms were used: “Internet AND use disorder (overuse OR addict* OR abuse OR use severity OR problematic OR dependence) AND (“Qol” OR “quality of life”).” Regarding PSU, the following terms were used: “smartphone (cellphone OR mobile phone) AND use disorder (overuse OR addict* OR abuse OR use severity OR problematic OR dependence) AND (“Qol” OR “quality of life”)”. The following number of articles were identified for PIU: Science Direct (438), PsycNET (221), and PubMed (549). The following number of articles were identified for PSU: Science Direct (102), PsycNET (1), and PubMed (46). Study selection was performed in two successive stages. First, the titles and abstracts of all potentially relevant articles were carefully scrutinized for eligibility according to inclusion criteria. Second, the full texts of the studies retained at the first stage were scrutinized for eligibility based on the same criteria. The PRISMA flowcharts illustrating the study selection process for each literature search are reported in Figs. 1 and 2.

### Data Extraction

The following data were extracted from the full text of the articles: country, sample size, age of the participants, study design, study goal, measurement instruments used for PIU or PSU and QOL or HRQOL, and study results. In the present study, we used the term “case-control” study to describe a study in which groups of participants were compared based on a pre-established criterion (e.g., the cutoff score on a specific scale). Information about content coverage (domains covered by the instruments) were extracted and analyzed. This information was used to establish content validity, that is, the extent to which a measure represents all facets of a given construct (i.e., sufficiently covers the measured construct). Poor content validity generally implies that the measurement instrument assesses too narrowly a construct. In our case, an instrument with poor content validity would be one that does not assess important aspects of QOL or HRQOL that might be affected by PIU or PSU.

## Results

This systematic review retained 17 studies for PIU that included 34,615 participants and 11 studies for PSU that included 204,118 participants. Six of 17 PIU studies (35.29%) and four of 11 PSU studies (36.36%) assessed HRQOL and the remainder of the studies examined QOL. All of the retained studies reported a negative correlation between QOL or HRQOL and PIU or PSU. The correlations reported in these studies ranged from *r* = −.13 to *r* = −.46 for PIU and *r* = −.09 to *r* = −.50 for PSU.

### Measurement Instruments Used to Assess QOL or HRQOL

The measurement instruments used in the retained studies differed in terms of the number of domains covered. Table 1 describes these instruments. Nine different psychometrically validated instruments were identified. A few instruments were composed of a series of items created in the context of a specific study and are not considered further in the present systematic review. The most used measurement instruments in PIU studies were the World Health Organization Quality of Life (WHOQOL) $(n=6),$ followed by the Pediatric Quality of Life Inventory (PedsQL) $(n=3)$ and the 12-item Short Form Survey (SF-12) $\left(n=3\right);$ the most used measurement instruments in PSU studies were the WHOQOL $(n=3)$ and the KIDSCREEN ($n=2$). None of the instruments used in the studies retained were specifically designed to assess QOL or HRQOL in the context of PIU or PSU.

**Table 1:** Description of quality of life (QOL) and health-related quality of life (HRQOL) instruments

Instrument	Target population	Number of items	Domains covered
**HRQOL scale**			
KIDSCREEN-27 [29]	Adolescents (aged 8-18)	27	Physical well-being, psychological well-being, autonomy, parental relationship, peer and social support, and school environment
24-h Migraine Quality of Life Questionnaire	Adults	15	Work functioning, social functioning, energy/vitality, migraine symptoms, and feelings/concerns
EQ-5D-3L [30]	Adults	5	Mobility, self-care, daily activities (e.g., work, study, housework, family, or leisure activity), anxiety/depression, and pain/discomfort
SF-12 [31]	Adults	12	Physical functioning, bodily pain, role limitations due to physical problems, general health vitality, social functioning, role limitations due to emotional problems, and perceived mental health
**QOL scale**			
WHOQOL-100 [21]	Adults	100	Physical health, mental health, social relationship, and environment
Pediatric Quality of Life Inventory [32]	Adolescents (aged 13–18)	23	Physical functioning, emotional functioning, social functioning, and school functioning
QOLS [33]	Adults	16	Material and physical well-being, relationships with other people, social, community and civic activities, personal development and fulfillment, and recreation
My Life as a Student questionnaire [34]	Adolescents (no age range specified)	26	School experience, opportunities to make autonomous decisions, relationships with classmates, current living conditions, family relationships, praise received when due, and availability of assistance
Subjective QOL questionnaire [35]	Adolescents (no age range specified)	47	Anxiety experience, depression experience, peer interaction, school life, family, somatosensory, and self-awareness

*EQ-5D-3L*, three-level EuroQoL-5 Dimension questionnaire; *QOLS*, Quality of Life Scale; *SF-12*, 12-item Short Form Survey; *WHOQOL-100*, World Health Organization Quality of Life assessment instrument

All domains assessed by QOL or HRQOL instruments in the retained studies are described in Tables 1 and 2.

**Table 2:** Domains explored by QOL or HRQOL in PIU or PSU

Domain	Subdomain	KIDSCREEN-27	24-h MQOLQ	EQ-5D-3L	SF-12	WHOQOL	PedsQL	QOLS	My Life as a Student questionnaire	Subjective QOL questionnaire
Physical health	Daily activities	×	×	×	×	×	×	×		
	Energy and fatigue	×	×	×	×	×	×			
	Bodily pain		×	×	×	×	×			
	Sleep and rest					×	×			
Psychological health	Negative and positive affect	×	×	×	×	×	×	×	×	×
	Bodily image and appearance					×				
	Loneliness	×				×				
	Memory and concentration	×				×	×			
Relations	Familial relations	×			×			×	×	×
	Social relations	×			×	×	×	×	×	×
School performance		×					×		×	×
Quality of environment	Physical environment							×		
	Financial problems	×				×		×		
	Security							×		
	Health and social care							×		
Satisfaction with life						×				

*EQ-5D-3L*, three-level EuroQoL-5 Dimension questionnaire; *HRQOL*, health-related quality of life; *24-h MQOLQ*, 24-h Migraine Quality of Life Questionnaire; *PedsQL*, Pediatric Quality of Life Inventory; *PIU*, problematic Internet use; *PSU*, problematic smartphone use; *QOL*, quality of life; *QOLS*, Quality of Life Scale; *SF-12*, 12-item Short Form Survey; *WHOQOL*, World Health Organization Quality of Life assessment instrument

The WHOQOL was the most frequently used instrument in the retained studies. Domains and facets incorporated in this comprehensive instrument include physical health (activities of daily living, dependence on medicinal substances and medical aids, energy and fatigue, mobility, pain and discomfort, sleep and rest, work capacity), psychological health (bodily image and appearance, negative affect, positive affect, self-esteem, spirituality/religion/personal beliefs, learning, memory and concentration), quality of social relationships (personal relationships, social support, sexual activity), and quality of environment (financial resources, freedom, physical safety and security, health and social care: accessibility and quality, home environment, opportunities for acquiring new information and skills, participation in and opportunities for recreation or leisure activities, physical environment [pollution/noise/traffic/climate], transport).

In order to synthetize and compare the results of the studies retained in the present systematic review, we reclassified the domains covered by the measurement instruments used into six different domains and 14 subdomains: physical health (daily activities, energy and fatigue, bodily pain, sleep and rest), psychological health (negative and positive effect, bodily image and appearance, loneliness, memory and concentration), relations (family relations, social relations), school performance, quality of environment (physical environment, financial problems, security, health and social care), and satisfaction with life, as illustrated in Table 2. This classification was conducted in order to identify specific categories for domains established as critical in the context of PIU and PSU, such as perceived loneliness or familial relationships.

An analysis of the domains covered by these instruments shows high heterogeneity. For instance, physical health is not assessed by the My Life as a Student questionnaire and the Subjective QOL questionnaire. Although all scales that we identified assessed psychological health, the specific aspects of psychological health that were measured differed among scales. For example, negative and positive affect (e.g., anxiety and depression) were assessed by almost all of the instruments, but loneliness was considered only in the KIDSCREEN and the WHOQOL.

### Measurement Instruments Used to Assess PIU and PSU

The studies included in the current systematic review mainly assessed PIU with Young’s Internet addiction test (YIAT) [36], Chen Internet addiction scale (CIAS) [37], and the Generalized Problematic Internet Use (GPIUS) [38]. For PSU, the most widely used scales were the smartphone addiction short version (SAS-SV) [39], the mobile phone problem use scale (MPPUS) [40], and the mobile phone addiction index (MPAI) [41]. These scales have been found to present with good psychometric properties [42, 43].

### Relationships Between PIU and QOL or HRQOL

Retained articles for PIU are synthesized in Table 3. Most studies that used the WHOQOL showed negative correlations between PIU and QOL or HRQOL domains [14, 44, 45]. Interestingly, a few studies considered more than a global score of QOL or HRQOL with the WHOQOL and reported that some domains are not linked to PIU, for example, the environmental domain [46, 47]. On the whole, studies that used the WHOQOL consistently showed that PIU is negatively correlated with QOL or HRQOL.

**Table 3:** Instruments and results of QOL or HRQOL in PIU studies

**Author**	**Country**	**Population**	**Age**	**Study design**	**Study goal**	**IA instrument**	**QOL instrument**	**Associations between PIU and QOL**
Barayan et al. (2018) [48]	Saudi Arabia	2516 female students	M_age_ = 21 Range = (17-25)	Correlational study	To assess the HRQOL factors and their relation to PIU	Changed form of Young's short version IA test	SF-12	PIU was negatively correlated with HRQOL domains related to mental and physical health
Cai et al. (2021) [44]	China	1070 nursing students	M_age_ = 19.7 (SD = 1.4)	Case-control comparison study	To determine the prevalence of PIU among baccalaureate nursing students	YIAT	WHOQOL	PIU was associated with lower QOL
Cam and Top (2020) [49]	Turkey	1558 high school students	M_age_ = 16.20 (SD = 1.05)	Correlational study	To investigate the prevalence of PIU among high school students, to evaluate its potential risk factors, and to investigate its relation to self-esteem and HRQOL	YIAT	SF-12	PIU was associated with both the physical and mental domains of HRQOL
Chern and Huang (2018) [14]	Taiwan	1439 college students	M_age_ = 20.51 (SD = 1.82)	Correlational study	To assess the association of PIU with lower HRQOL	26-item CIAS	WHOQOL	PIU individuals had significantly lower HRQOL
Cruz et al. (2018) [50]	Brazil	254 high school students	M_age_ = 15.1 (SD = 1.3)	Case-control comparison study	To evaluate PIU and QOL	YIAT	PedsQL	Students with PIU had a lower mean quality of life in the physical, emotional, social, and educational aspects
Eliacik et al. (2016) [51]	Turkey	71 obese adolescents + 64 control group subjects	Not reported	Case-control comparison study	To evaluate PIU, sleep, and HRQOL among obese individuals	IAS	PedsQL	Adolescents with PIU had poorer HRQOL
Fatehi et al. (2016) [46]	Iran	174 undergraduate medical students	M_age_ = 22.57 (SD = 1.24)	Case-control comparison study	To examine QOL in medical students with PIU	YIAT	WHOQOL	QOL was lower in medical students with PIU (based on the YIAT)
Tingting et al. (2019) [52]	China	701	M_age_ = 20.50 (SD = 1.42)	Correlational study	To evaluate the effects of PIU on QOL in college students	Not reported	Note reported	PIU was negatively correlated with QOL
Gao et al. (2020) [53]	Germany	446	M_age_ = 25.8 (SD = 11.6)	Case-control comparison study	To evaluate the relationship of work-time and leisure-time Internet use with PIU and QOL	PIU measured on a 4-point Likert scale with 10 items	WHOQOL	Results showed a negative relationship between PIU and perceived QOL
Huang et al. (2020) [54]	China	12,507	M_age_ = 16.6 (SD = 0.8)	Case-control comparison study	To evaluate the relationships between PIU and suicidal ideation, mood disorders, QOL, and personality traits among adolescents	IAT	The QOL questionnaire by Xie [55]	An association between PIU and QOL was reported
Karimy et al. (2020) [56]	Iran	279	M_age_ = 21.01 (SD ± 3.17)	Case-control comparison study	To evaluate the association between PIU, sleep quality, and HRQOL	YIAT	SF-12	Students with moderate to severe PIU (based on the YIAT) had poorer HRQOL in both psychological and physical domains except for physical pain
Lu et al. (2018) [47]	China	1385 students	Not reported	Case-control comparison study	To assess the prevalence of PIU and its association with QOL	YIAT	WHOQOL	Students with PIU (based on the YIAT) had significantly lower QOL in physical, psychological, and environmental domains but not in social domains
Machimbarrena et al. (2019) [57]	Spain	12,285	M_age_ = 14.69 (SD = 1.73)	Correlational study	To assess the impact of PIU on adolescent HRQOL	GPIUS2	KIDSCREEN-27	A negative relationship between PIU and HRQOL was reported
Pontes et al. (2015) [58]	England	1057	M_age_ = 30 (SD = 10.84) Range = 16–70	Correlational study	To evaluate the impacts of Internet-based specific activities on the perceptions of Internet addiction, QOL, and excessive usage	A single online questionnaire	A single online questionnaire	PIU (reflected by perceived dependence toward Internet use) was correlated with lower QOL
Takahashi et al. (2018) [59]	Japan	7857 elementary + 4600 junior high school students	Not reported	Case-control comparison study	To assess the prevalence of PIU and its association with depression and HRQOL	YDQ	PedsQL	PIU was negatively correlated with physical, emotional, and school functioning domains of HRQOL, whereas this association was not significant in the social functioning domain
Tran et al. (2017) [16]	Vietnam	566	Range = 15–25	Correlational study	To evaluate the influence of PIU and online interpersonal influences on HRQOL	YIAT	EQ-5D-5L and EQ-VAS	PIU was significantly associated with lower HRQOL
Xu et al. (2020) [45]	China	2892 secondary school students	M_age_ = 15.1 (SD = 1.7)	Case-control comparison study	To compare the prevalence of PIU among adolescents between Macau and mainland China and to examine its association with QOL	IAT	WHOQOL	Students with PIU (based on the IAT) reported lower quality of life in physical, psychological, social, and environmental domains

*CIAS*, Chen Internet Addiction Scale; *EQ-5D-5L*, five-level EuroQoL-5 Dimension questionnaire; *EQ-VAS*, EuroQoL Visual Analogue Scale; *GPIUS2*, Generalized Problematic Internet Use Scale 2; *HRQOL*, health-related quality of life; *IA*, Internet addiction; *IAS*, Internet Addiction Scale; *IAT*, Internet Addiction Test; *PedsQL*, Pediatric Quality of Life Inventory; *PIU*, problematic Internet use; *QOL*, quality of life; *SF-12*, 12-item Short Form Survey; *WHOQOL*, World Health Organization Quality of Life assessment instrument; *YDQ*, Young’s Diagnostic Questionnaire; *YIAT*, Young Internet Addiction Test

Studies conducted with other instruments globally reproduced the same patterns of results. Studies that used the PedsQL generally showed a negative association with domains of the HRQOL or QOL [48, 49]. Yet, a study by Cruz et al. [50] found no correlation with social functioning. Studies that used the SF-12 showed that physical and psychological domains are both affected [51, 56], except for physical pain [59].

### Relationships Between PSU and QOL or HRQOL

The articles retained for PSU are synthesized in Table 4. All of these studies reported a negative correlation between PSU and QOL or HRQOL. Studies that considered the various domains assessed by the WHOQOL showed that PSU is negatively correlated to all QOL domains assessed [60] and that the psychological domain is most affected [61].

**Table 4:** Instruments and results of QOL or HRQOL in PSU studies

**Author**	**Country**	**Population**	**Age**	**Study design**	**Study goal**	**Smartphone use instrument**	**QOL instrument**	**Associations between PSU and QOL**
Awasthi et al. (2020) [61]	India	395 medical students	Range= 21–24	Case-control comparison study	To assess PSU and its association with the QOL of medical students	SAS-SV	WHOQOL – BREF	PSU was high among medical students and negatively correlated with all assessed domains of QOL
Buctot et al. (2020) [17]	Philippines	1447 high school students	Range= 13–18	Correlational study	To investigate the relationship between PSU and HRQOL	SAS-SV	KIDSCREEN-27	A negative association between PSU and total HRQOL was found, as well as its subdomains physical well-being, psychological well-being, and school environment, but not with autonomy, parents, peers, or social support
Demir and Sumer (2019) [62]	Turkey	123 patients	Range= 18–65	Case-control comparison study	To investigate the effects of PSU on headache, sleep quality, daytime sleepiness, and QOL in migraine patients	MPPUS	24-h MQOLQ	A negative correlation between PSU and 24-h MQOLQ was reported
Gao et al. (2017) [63]	China	722 Chinese university students	M_age_ = 20.50 (SD = 1.42) Range= 16–25	Correlational study	To investigate the mediating effect of PSU and depression on QOL	MPAS	WHOQOL	PSU was negatively correlated with QOL
Gao et al. (2020) [64]	China	1767	M_age_ = 13.33 (SD = 1.94) Range= 10–18	Correlational study	To evaluate the effects of the parent-child relationship on PSU and the mediating role of QOL	MPAI	Subjective QOL questionnaire	Adolescent PSU negatively predicted QOL
Hughes and Burke (2018) [65]	UK	95	Range = 22–73	Case-control comparison study	To assess the impact of overnight smartphone use on well-being	SAS-SV	QOLS	People who restricted bedtime smartphone use reported higher QOL
Jeong et al. (2020) [66]	Korea	190,066	Range= 919–80	Case-control comparison study	To evaluate the relationship of the frequency of impairments in daily activities due to PSU with HRQOL	A question was asked to assess problematic use	EQ-5D-3L	Groups of participants considered to have more severe PSU were characterized by lower HRQOL
Li et al. (2020) [60]	China	2312	18 and above	Case-control comparison study	To evaluate PSU and its relationship with QOL	MPAS	WHOQOL	Students characterized as addicted to their mobile phones (based on MPAS) had significantly lower QOL in all domains
Mascia et al. (2020) [67]	Italy	215 students	M_age_ 12.7 (SD = 0.90)	Correlational study	To assess the relationship of emotional intelligence, self-regulation, and PSU with student well-being and QOL	SAS	My Life as a Student questionnaire	PSU was found to act as a mediator in the relationship between self-regulation and well-being
Mireku et al. (2019) [68]	UK	6616 students	Range= 11–12	Correlational study	To investigate the relationship of night-time screen-based media devices use with HRQOL	A computer-based assessment	KIDSCREEN-10	Night-time use of mobile phones was associated with lower HRQOL
Miri et al. (2020) [69]	Iran	360 medical students	M_age_ = (25.1–6.3)	Correlational study	To assess the relationship of PSU with QOL in medical students	PMPAS questionnaire	SF-12	The prevalence of PSU negatively correlated with QOL of students and had a significant negative relationship with mental function, whereas this relationship was not significant for physical function

*EQ-5D-3L*, three-level EuroQoL-5 Dimension questionnaire; *HRQOL*, health-related quality of life; *MPAI*, Mobile Phone Addiction Index; *MPAS*, Mobile Phone Addiction Scale; *MPPUS*, Mobile Phone Problematic Use Scale; *24-h MQOLQ*, 24-h Migraine Quality of Life Questionnaire; *PSU*, problematic smartphone use; *PMPAS*, Mobile Phone Addiction Scale; *QOL*, quality of life; *QOLS*, Quality of Life Scale; *SAS*, Smartphone Addiction Scale; *SAS-SV*, Smartphone Addiction Scale – Short Version; *SF-12*, 12-item Short Form Survey; *WHOQOL*, World Health Organization Quality of Life assessment instrument; *WHOQOL – BREF*, World Health Organization Quality of Life assessment instrument; *YDQ*, Young’s Diagnostic Questionnaire; *YIAT*, Young Internet Addiction Test

Buctot and colleagues [17] also showed, using KIDSCREEN-27, that PSU is negatively correlated with several domains (physical health and psychological health, school environment) but unrelated to others (e.g., autonomy, parental, and peer support). Another study that used the SF-12 showed that PSU is associated with poor mental health but not physical health [69].

## Discussion

In this systematic review, we synthesized the studies that explored the relationships between PIU or PSU and QOL or HRQOL and critically evaluated the measurement instruments used to assess QOL or HRQOL in these studies. Addressing this topic is warranted, as a substantial part of previous research explored PIU and PSU while not necessarily considering the negative consequences associated with Internet or smartphone use, thus potentially overpathologizing normal technology use [12••].

Here, we provided a summary of the measurement instruments used to assess QOL or HRQOL in existing studies and examined their content validity in the context of PIU and PSU. It might be that QOL and HRQOL instruments not specifically developed in the context of PIU and PSU research do not include domains that are particularly relevant to these problematic behaviors. Our analysis showed that there was an important heterogeneity in the domains covered by QOL and HRQOL instruments used in the retained studies. Moreover, different instruments can assess similar domains with diverging items, thus further complicating the comparison among studies. For example, four of the nine identified instruments (i.e., KIDSCREEN, PedsQL, My Life as a Student questionnaire, and the Subjective QOL questionnaire) evaluated the school domain, but with different items and concepts.

This review shows that WHOQOL for adults and PedsQL, which targets participants aged 13–18 years, are the most used measurement instruments. In terms of the classification of domains in the current systematic review, these instruments cover physical health, psychological health, social relations, quality of the environment, and satisfaction with life (WHOQOL), or physical health, psychological health, social relations, and school performance (PedsQL), making them the most convenient instruments at hand. Notably, some of the instruments identified in this systematic review and synthesized in Table 1 (including the most used instrument: WHOQOL) do not cover key domains such as familial relations, which is a crucial variable regarding PIU [70, 71]. Despite the content-coverage limitation described earlier, the QOL and HRQOL instruments used in the retained studies could be effective in measuring a change after intervention [72]. Clinical studies are indeed lacking, and it would be interesting to determine which domains of QOL and HRQOL might be affected by treatment programs or preventive actions. Further research could also overcome the content coverage problem identified in the current study with the development and validation of new instruments, potentially based on qualitative analysis conducted in individuals with PIU or PSU that has clear negative consequences and causes functional impairment.

All retained studies reported negative correlations between QOL or HRQOL and PIU or PSU. The majority of studies were published within the last 3 years, indicating recent research interest, likely fueled by clinical demand or by the recognition that PIU and PSU have become internationally relevant public health issues [3]. On the whole, existing evidence indicates a significant negative relationship between PIU or PSU and the psychological and physical domains of QOL or HRQOL, which is in line with a recent review of these relationships in the context of the COVID-19 pandemic [73]. However, the heterogeneity of the instruments used makes it difficult to compare other affected domains in the retained studies, such as environmental and social domains [14, 46, 47, 52, 74].

This systematic review comes with several limitations. First, the number of studies was relatively limited, and most were cross-sectional studies conducted with self-selected participants, thus hindering causal interpretation and compromising the representativeness of the findings. Given the public health relevance of technology-mediated problematic behaviors, future research in this field should be conducted in nationally representative samples or should follow longitudinal designs. Moreover, few studies have surveyed clinical participants or tested the impact of prevention or treatment approaches on QOL or HRQOL. Second, we considered only studies written in English, and it is possible that relevant literature published in other languages was neglected. Much research published in national East-Asian journals (e.g., Japanese, Korean, or Chinese journals) could have been relevant to the topic under study (see, e.g., Long et al. [75], for the necessity of considering such literature in the context of technology-mediated problematic behaviors). Third, most of the retained studies reported an overall correlation between PIU or PSU and QOL or HRQOL without giving detailed information on the specific domains affected. Fourth, recent research suggests that the terms “Internet addiction” and “smartphone addiction” might be deceptive. Indeed, these terms are umbrella constructs that encompass a wide range of potentially problematic technologically mediated behaviors involving various online activities [18••, 76, 77, 13], for which the Internet or a smartphone serves as the common vector or “delivery mechanism” [78, 79, 80]. According to these views, the focus must be on specific online activities, not on the medium through which they take place. Yet, for parsimony reasons, we decided not to include studies focusing on specific online activities (e.g., videogames or social network sites). This could have resulted in excluding potential relevant studies about QOL/HQOL and specific problematic online behaviors. Accordingly, it would be important to consider the evidence linking specific problematic online behaviors and QOL or HRQOL in future systematic literature reviews. That being said, it was also proposed that technology-mediated problematic behaviors are to be conceptualized within a spectrum of related disorders associated with both common and unique etiological factors [6, 7], implying that an analysis of their commonalities, as was done in the current systematic review, is also required.

## Conclusion

Because of the widespread prevalence of PIU and PSU, which tend to be linked with lower QOL or HRQOL, in particular poor mental and physical health, promotion of a more systematic public health campaign is required to target the healthy use of these communication devices. Prevention programs should also target vulnerable individuals, focusing on the most affected domains of QOL or HRQOL (i.e., physical and psychological health). Among existing instruments, WHOQOL for adults and PedsQL for adolescents (aged 13–18 years) proved to be the most relevant, as shown from the results of this systematic review, although there is a need for new measurement instruments that target domains that are specifically relevant in the context of PIU and PSU (e.g., specific physical and psychological health domains such as sleep and loneliness, quality of familial relations).
